# Ancient Grandeur of the Vertebrate Neuropeptide Y System Shown by the Coelacanth *Latimeria chalumnae*

**DOI:** 10.3389/fnins.2013.00027

**Published:** 2013-03-08

**Authors:** Dan Larhammar, Christina A. Bergqvist

**Affiliations:** ^1^Unit of Pharmacology, Department of Neuroscience, Science for Life Laboratory – Uppsala UniversityUppsala, Sweden

**Keywords:** G-protein-coupled receptor, neuropeptide Y, peptide YY, pancreatic polypeptide, coelacanth, *Latimeria chalumnae*

## Abstract

The neuropeptide Y (NPY) family receptors and peptides have previously been characterized in several tetrapods, teleost fishes, and in a holocephalan cartilaginous fish. This has shown that the ancestral NPY system in the jawed vertebrates consisted of the peptides NPY and peptide YY (PYY) and seven G-protein-coupled receptors named Y1–Y8 (Y3 does not exist). The different vertebrate lineages have subsequently lost or gained a few receptor genes. For instance, the human genome has lost three of the seven receptors while the zebrafish has lost two and gained two receptor genes. Here we describe the NPY system of a representative of an early diverging lineage among the sarcopterygians, the West Indian Ocean coelacanth *Latimeria chalumnae*. The coelacanth was found to have retained all seven receptors from the ancestral jawed vertebrate. The receptors display the typical characteristics found in other vertebrates. Interestingly, the coelacanth was found to have the local duplicate of the PYY gene, called pancreatic polypeptide, previously only identified in tetrapods. Thus, this duplication took place very early in the sarcopterygian lineage, before the origin of tetrapods. These findings confirm the ancient complexity of the NPY system and show that mammals have lost more NPY receptors than any other vertebrate lineage. The coelacanth has all three peptides found in tetrapods and has retained the ancestral jawed vertebrate receptor repertoire with neither gains or losses.

## Introduction

Neuropeptide Y (NPY) and its related peptides named peptide YY (PYY) and pancreatic polypeptide (PP) comprise a system of neuronal and endocrine peptides that act on several G-protein-coupled receptors in vertebrates. They are involved in the regulation of a broad range of functions including appetite/satiety, gut motility, cardiovascular activity, pituitary release of hormones, circadian rhythm, and many more (see Pedrazzini et al., 2003; Brumovsky et al., 2007; Mercer et al., 2011; Zhang et al., 2011 for reviews). NPY and PYY arose by duplication of a common ancestral peptide gene before the vertebrate radiation (Larhammar, 1996). In mammals NPY is almost exclusively neuronal whereas PYY is primarily expressed in endocrine cells in the gastrointestinal tract. In ray finned fishes, PYY too is expressed in the nervous system (Cerdá-Reverter et al., 2000; Söderberg et al., 2000). PP is a local duplicate of PYY (Hort et al., 1995) previously found only in tetrapods (Larhammar, 1996; Cerdá-Reverter and Larhammar, 2000; Conlon, 2002; Sundstrom et al., 2008) and is expressed in pancreatic islets.

Mammals generally possess four receptors for the NPY-family peptides, namely subtypes Y1, Y2, Y4, and Y5 (Larhammar and Salaneck, 2004). Subtype Y3 was postulated from pharmacological experiments but does not exist as a separate gene (Herzog et al., 1993; Jazin et al., 1993). A fifth receptor gene, Y6, is expressed in a few mammals such as mouse and rabbit but is a pseudogene in many others including primates (Matsumoto et al., 1996), pig (Wraith et al., 2000), and guinea-pig (Starbäck et al., 2000). With its three peptides and four receptors, the NPY system in humans and most other mammals displays a degree of complexity resembling many other vertebrate peptide-receptor systems for neuronal and endocrine peptides, for instance the melanocortin system (Dores and Baron, 2011; Liang et al., 2013), the opioid system (Sundstrom et al., 2010), the oxytocin-vasopressin system (Ocampo Daza et al., 2011; Yamaguchi et al., 2012), and the somatostatin-cortistatin system (Tostivint et al., 2008; Ocampo Daza et al., 2012). However, previous evolutionary studies of the NPY receptor family have shown that a larger number of receptors existed in the early stages of vertebrate evolution before the emergence of jawed vertebrates, *Gnathostomata*: by sequence-based phylogenetic analyses and comparison of gene locations on chromosomes, we were able to deduce an ancestral vertebrate set of no less than seven NPY-family receptors (Larhammar and Salaneck, 2004; Larsson et al., 2008), more than for any other known peptide-receptor family. Subsequently, this repertoire was confirmed by our identification of all seven receptor subtypes in a cartilaginous fish (*Chondrichthyes*), the holocephalan elephant shark, or ghost shark, *Callorhinchus milii* (Larsson et al., 2009). Thus, the following evolutionary scenario was corroborated: an ancestral pre-vertebrate chromosome carried the genes for a Y1-like, a Y2-like, and a Y5-like receptor subtype. The two basal vertebrate tetraploidizations (Nakatani et al., 2007; Putnam et al., 2008) quadrupled this chromosome, thereby generating four similar chromosomal regions that probably had as many as 12 (4 × 3) family members, unless some were lost already after the first tetraploidization. In extant vertebrate lineages a total of seven family members have been found to remain: four Y1-subfamily genes with one on each of the four chromosomes resulting from the tetraploidizations (Y1, Y4, Y6, and Y8), two Y2-like genes (Y2 linked to Y1, Y7 linked to Y6), and a single surviving Y5 gene (linked to Y1 and Y2) (Larsson et al., 2008, 2009).

Of the vertebrates investigated to date only the elephant shark has maintained all seven of these ancestral vertebrate receptor genes (Larsson et al., 2009). All of the other gnathostome lineages seem to have suffered losses, although some of the genome databases may be incomplete. Among amphibians, the western clawed frog *Silurana (Xenopus) tropicalis* seems to have lost Y6 which appears to be a pseudogene in the frog *Pelophylax esculentus* (previously called *Rana esculenta*; Sundstrom et al., 2012). Amniotes have lost Y8, or possibly this gene was lost independently in birds and mammals (Larsson et al., 2009). The mammalian lineage subsequently lost also Y7. The Y6 gene is a pseudogene in several mammals as mentioned above. In the large and heterogeneous group of rayfinned fishes (*Actinopterygii*), the teleosts are the most carefully studied. In the true teleost species (*Euteleostei*) with sequenced genomes, the receptors Y1, Y2, Y4, Y7, and Y8, as well as a duplicate of Y8 called Y8b, have been identified, albeit only the zebrafish genome contains all of these (Larsson et al., 2008; Salaneck et al., 2008). Thus, the Y8 duplicate seems to be the only surviving copy resulting from the teleost-specific third tetraploidization, 3R (Jaillon et al., 2004). Also, this suggests that Y5 and Y6 are missing in *Euteleostei*. A local duplicate of Y2, named Y2-2, has been found in zebrafish and medaka (Fallmar et al., 2011). In addition, Y5 and Y6 have been identified in more basally diverging rayfinned fish species, namely a sturgeon and a bichir, as well as an early diverging teleost, the silver arowana, *Osteoglossum bicirrhosum* (Salaneck et al., 2008). Also, we have previously cloned the genes for Y5 and Y6 from the coelacanth *Latimeria chalumnae* (Lch; Larsson et al., 2007). The receptor gene duplication scenario is further supported by findings in lampreys of members of the Y1, Y2, and Y5 lineages (Salaneck et al., 2001; Larsson et al., 2009; Xu et al., 2012). In summary, of all gnathostome species that have been previously investigated only the elephant shark seems to have retained the complete ancestral repertoire of seven NPY receptor genes.

The West Indian Ocean coelacanth, Lch, is an important species for studies of vertebrate evolution as it is one of only two extant and closely related coelacanth species representing a very early *Actinistia* branch among the lobe finned fishes, *Sarcopterygii*. The relationship of coelacanths to lungfishes (Dipnoi) and tetrapods has been difficult to resolve. Recent data suggest that the coelacanths are slightly more closely related to the lungfishes than either of these groups is to tetrapods (Shan and Gras, 2011). The Actinopterygii-Sarcopterygii divergence took place approximately 424 million years ago (Mya) according to a recent estimate based on molecular data (Chen et al., 2012). The coelacanth and tetrapod lineages may have parted only a few tens of millions of years later, well before the split of the amphibian and amniote lineages approximately 350 Mya. Thus, it is of great interest to see which genetic and phenotypic Sarcopterygian characters had already arisen before the coelacanth-tetrapod divergence. In September 2011, a new genome assembly became available for the coelacanth. We present here a genomic analysis of the NPY system in Lch, identifying not only the receptor repertoire but also the genes for the three peptide ligands that bind to these receptors.

## Materials and Methods

The PreEnsembl sequence database (ENSEMBL LatCha1 released September 2011, database version 69.1) for the West Indian Ocean coelacanth Lch was searched with Blast/Blat using as query sequences the human NPY-family receptors Y1, Y2, and Y4, and the zebrafish receptors Y7 and 8b. Five new receptors were identified, all but Y4 are annotated in Ensembl68. Amino acid sequences for NPY receptors from the following species were used for alignments: human (*Homo sapiens*), chicken (*Gallus gallus*), zebrafish (*Danio rerio*), elephant shark (*C. milii*), mouse (*Mus musculus*), and coelacanth (*L. chalumnae*). A list of genes with corresponding accession numbers is provided in Table 1 with the coelacanth NPY receptor sequences in bold.

**Table 1:** **The table lists Ensemble gene ID or NCBI accession number for the coelacanth NPY-family receptor and peptide genes as well as the orthologs genes in human, chicken, zebrafish, and elephant shark**.

Species	Receptor	Ensembl gene ID/NCBI accession number	Genome assembly	Comment
**Coelacanth**	**Y1**	**ENSLACG00000009650**	LatCha1	Manually curated
Human	Y1	NM_000909		
Chicken	Y1	NM_001031535		
Zebrafish	Y1	EU046342		
Elephant shark	Y1	EU637847		
**Coelacanth**	**Y2**	**ENSLACG00000009115**	LatCha1	Manually curated
Human	Y2	NM_000910		
Chicken	Y2	NM_001031128		
Zebrafish	Y2	XP_001343301		
Zebrafish	Y2-2	XP_001332759		
Elephant shark	Y2	EU637848		
**Coelacanth**	**Y4**	**Not annotated in database** (JH127692.1: 3,965–4,993)		
Human	Y4	NM_005972		
Chicken	Y4	AF410853		
Zebrafish	Y4	AF037400		
Elephant shark	Y4	EU637849		
**Coelacanth**	**Y5**	**ABI94072.1**		
Human	Y5	NM_006174		
Chicken	Y5	NM_001031130		
Elephant shark	Y5	EU637850		
**Coelacanth**	**Y6**	**ABI94073.1**		
Mouse	Y6	ENSMUSG00000038071	GRCm38	
Chicken	Y6	ENSGALG00000021235	WASHUC2	Manually curated
Elephant shark	Y6	EU637851		
**Coelacanth**	**Y7**	**ENSLACG00000013918**	LatCha1	Manually curated
Chicken	Y7	NP_001032913		
Zebrafish	Y7	AY585098		
Elephant shark	Y7	EU637852		
**Coelacanth**	**Y8**	**ENSLACG00000008470**	LatCha1	
Zebrafish	Y8a	NM_131437		
Zebrafish	Y8b	AF030245		
Elephant shark	Y8	EU637853		
Human	SSTR1	ENSG00000139874	GRCh37	

*For Y6, the mouse sequence is included instead of the human pseudogene. The human somatostatin receptor SSTR1 that was used as outgroup in the phylogenetic tree is also included*.

*The coelacanth entries are shown in bold*.

Amino acid alignments were made in Jalview 2.8 version 14.0 (Waterhouse et al., 2009) using the MUSCLE and Clustal W web tool with standard settings. Phylogenetic Neighbor-Joining (NJ; Saitou and Nei, 1987) trees were made by using Clustal X version 2.0.12 (Larkin et al., 2007), standard settings and 1000 bootstrap replicates were applied. The tree shown in Figure 2 was rooted with the human somatostatin receptor sequence SSTR1. Phylogenetic trees were also made with the Maximum Likelihood (ML) method (Guindon et al., 2010).

## Results

### *Latimeria chalumnae* NPY-family receptors

We have previously reported the coelacanth Y5 and Y6 genes (Larsson et al., 2007). To identify additional NPY-family receptors, we searched the PreEnsemble Lch database with sequences from other species as queries. The identified Lch sequences were aligned with known NPY-family receptors from other vertebrates and subjected to phylogenetic analyses. Some of the sequences had to be manually curated as indicated in Table 1 which lists all the identified Lch sequences and their Ensemble ID or NCBI accession numbers. The table also lists the sequences for the other species that are included in the alignment and the phylogenetic tree described below. The searches allowed us to identify all the NPY-family receptors known to have arisen before the divergence of the jawed vertebrates, i.e., also Y1, Y2, Y4, Y7, and Y8.

**Figure 1:**
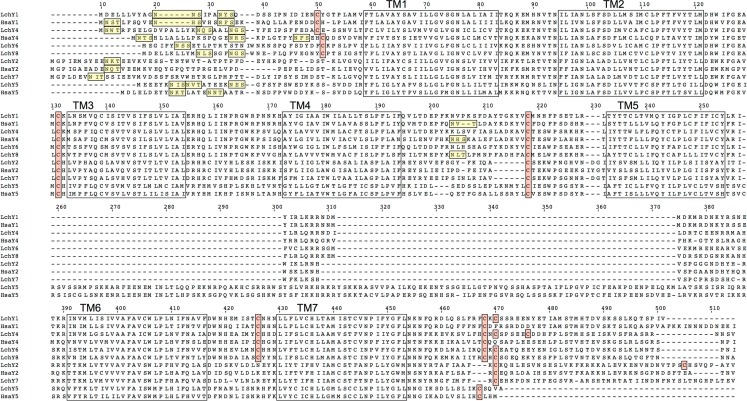
**Alignment of all coelacanth and human NPY-family receptor sequences except the human hY6 pseudogene**. The seven transmembrane regions are marked with boxes. Consensus sequences for asparagine-linked glycosylation in the aminoterminal region are underlined. Cysteines assumed to be involved in disulfide bonds are within boxes as are the cysteines in the carboxyterminal region that may serve as attachment sites for palmitate.

An alignment of all Lch and human NPY-family receptors is shown in Figure 1. All of the sequences contain one to three consensus sequences for *N*-linked glycosylation in the aminoterminal region of the receptors, before transmembrane region 1 (TM1), i.e., the sequence NXS/T. The Lch Y6 and Y8 sequences also have a consensus glycosylation sequence in extracellular loop 2 (EL2), as do human Y1 and Y4. All sequences have a cysteine residue in EL1 and one cysteine in EL2 that presumably form a disulfide bond. The four Y1-subfamily sequences Y1, Y4, Y6, and Y8 also have a cysteine in the aminoterminal region and one in EL3, expected to form an additional disulfide bond, as in Y1-subfamily sequences from other species. Finally, one ore more cysteines are present in the cytoplasmic tail after TM7, expected to serve as attachment sites for palmitate to anchor the tail to the inner side of the cytoplasmic membrane. Also many other positions that are known to be highly conserved among NPY-family receptors, either between species or between receptor subtypes (or both), are conserved in the Lch sequences.

**Figure 2:**
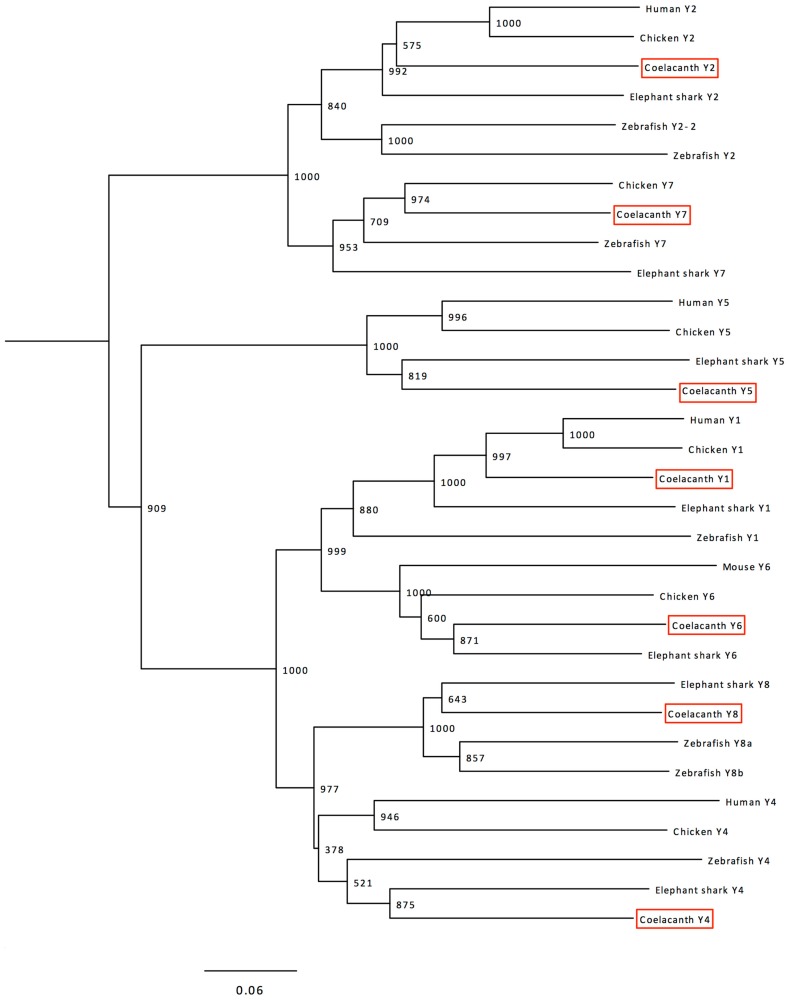
**Phylogenetic tree calculated with the Neighbor-Joining method showing the relationships of the NPY-family receptor sequences in coelacanth, human (except Y6 where the mouse sequence is included instead of the human pseudogene), chicken, zebrafish, and elephant shark**. The human somatostatin receptor 1 (SSTR1) was used as outgroup to root the tree. The tree was calculated using the complete sequences aligned with Clustal W. Trees were also deduced using the Maximum Likelihood method and both methods were also used to calculate trees based on an alignment generated with MUSCLE. These trees are available from the authors upon request. See text for details.

Phylogenetic analyses were performed with a more extensive alignment that included also the sequences of the six chicken receptors and the seven elephant shark receptors, plus the mouse Y6 sequence as the human Y6 gene is a pseudogene. The resulting tree is shown in Figure 2. The seven Lch sequences could be clearly identified as orthologs to each of the seven previously described receptors in jawed vertebrates. The tree has a few minor deviations from the established order of divergence for the included species, mostly because of higher evolutionary rates for some lineages as explained in the discussion.

**Figure 3:**
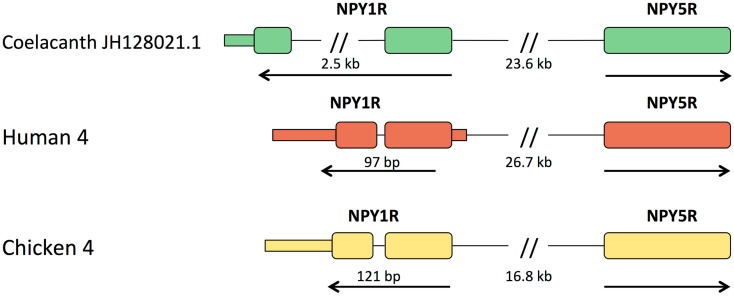
**Organization of the Y1 and Y5 genes relative to one another in coelacanth, human, and chicken**. Exons are shown as tall boxes for the coding region and low boxes for the 5′- and 3′-untranslated regions where known. The 3′-untranslated region is shown until the position of the first consensus poly(A) signal. The size of the intron in the coding region of Y1 is shown. In all three species, the genes have a “head-to-head” orientation and may share regulatory upstream elements. The distance between the coding regions of the two genes are shown, but it has not yet been fully explored where promoters and any separate 5′-untranslated exons may be located.

In all other vertebrates with assembled genomes, the genes for Y1 and Y5 are located close together in a head-to-head orientation, implying that their promoter regions may share some regulatory elements. The distance between the start codons for the human genes is 26.7 kb and for the chicken genes 16.8 kb. In the coelacanth, the distance is 23.6 kb (Figure 3). The promoter regions have not been characterized in detail for Y1 and Y5 in any species and furthermore seem to vary between species with regard to the possibility of multiple promoters and alternatively spliced 5′UTR exons (Wraith, 1999), why a detailed comparison with Lch is not meaningful at this point. The Y1 gene is the only member of the NPY receptor family that has an intron in the coding region. This intron is very small in mammals (97 bp in the human gene, 110 bp in opossum), chicken (121 bp), anole lizard (698 bp), and the frog *Silurana tropicalis* (92 bp; Sundstrom et al., 2012), but considerably larger in zebrafish with approximately 40 kb (in preparation), a sturgeon (Salaneck et al., 2008), and the two cartilaginous fishes spiny dogfish (Salaneck et al., 2003) and elephant shark (Larsson et al., 2009), the last-mentioned having an intron >3 kb. In the coelacanth, this intron is approximately 2.5 kb (Figure 3). Thus, the intron is large both in cartilaginous fishes, a teleost, and the coelacanth. The most parsimonious interpretation is that large size is ancestral and that the intron contracted in the ancestor of tetrapods and has remained small in this lineage.

The synteny groups of the NPY-family receptor genes and their neighboring genes in the same chromosome regions could not be analyzed in the coelacanth due to the small size of the scaffolds as a result of low-coverage sequencing.

### *Latimeria chalumnae* NPY-family peptides

The coelacanth genome database was searched with the three human members of the NPY peptide family as queries. Hits were found for the most highly conserved parts of both NPY, PYY, and somewhat surprisingly also PP, although this peptide has not previously been identified outside of the tetrapods. Further analyses revealed the presence of the two small 3′ exons encoding the carboxyterminal extensions of the prepropeptides for NPY and PYY. However, for the more rapidly evolving PP, these exons could not be found, most probably because they are missing in the Lch assembly. The Ensemble gene IDs are listed in Table 2.

**Table 2:** **The table lists Ensemble gene ID for the coelacanth and human NPY-family peptides NPY, PYY, and PP (pancreatic polypeptide)**.

Species	Peptide	Ensembl gene ID	Genome assembly	Comment
**Coelacanth**	NPY	ENSLACG00000001891	LatCha1	
Human	NPY	ENSG00000131096	GRCh37	
**Coelacanth**	PYY	ENSLACG00000015096	LatCha1	
Human	PYY	ENSG00000131096	GRCh37	
**Coelacanth**	Pancreatic polypeptide	**Not annotated in database** (JH126680.1:1,154,715–1,154,819)		
Human	Pancreatic polypeptide	ENSG00000108849	GRCh37	

*The coelacanth entries are shown in bold*.

**Figure 4:**
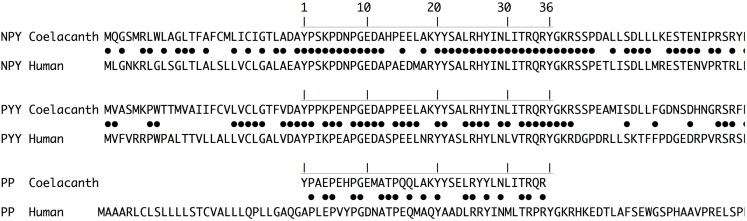
**Pairwise prepropeptide sequence alignments between coelacanth and human for NPY, PYY, and PP (pancreatic polypeptide)**. Identical positions are marked with dots for each pairwise comparison. The mature peptide sequences are marked with the horizontal line and the positions are numbered 1 through 36.

An alignment of the coelacanth and human prepropeptides is shown in Figure 4. The mature NPY sequences share 31 och the 36 positions and three of the remaining five are highly conservative replacements, as has been observed for NPY for other vertebrates (Larhammar, 1996; Cerdá-Reverter and Larhammar, 2000). The mature Lch and human PYY sequences share 27 of the 36 positions. The coelacanth PYY sequence is identical to the deduced ancestral vertebrate PYY sequence (not shown), thus the differences are due to divergence of PYY in the lineage leading to humans and other mammals. In contrast, PP has diverged considerably between coelacanth and human and the two species share only 18 of the 36 residues, a degree of identity found also for comparisons between birds or frogs and mammals (Larhammar, 1996; Cerdá-Reverter and Larhammar, 2000; Conlon, 2002). For both NPY and PYY, the differences that exist between coelacanth and human are predominantly located in the middle part of the peptides, suggesting that both ends are kept conserved for functional reasons, presumably interactions with the NPY-family receptors.

**Figure 5:**
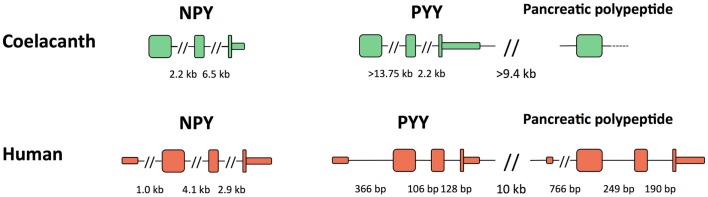
**Exon organization of the three coelacanth NPY-family peptide genes and their orthologs human peptide genes**. All genes are shown in the 5′-to-3′ direction. PYY and pancreatic polypeptide are located adjacent to each other and have the same orientation on the chromosome, thus a “head-to-tail” orientation. Exons are shown as tall boxes for the coding region and low boxes for the 5′- and 3′-untranslated regions where known. The 3′-untranslated region is shown until the position of the first consensus poly(A) signal. For the coelacanth genes, the separate 5′-untranslated exons are still unknown as are the two 3′ coding exons of the pancreatic polypeptide genes due to incomplete sequence information. Gaps in the genomic sequence of the coelacanth mean that some of its intron sizes and the intergenic distance are minimum estimates.

The genes for Lch NPY and PYY have the coding region distributed over three exons like in all other vertebrates (Figure 5). The introns in the coelacanth PYY gene are much larger, >13.75 and 2.2 kb, respectively, than in the mammalian orthologs, with only 106 and 128 bp in human. The introns in chicken and zebrafish are of intermediate size with 2.2 and 0.3 kb in chicken and similar sizes in zebrafish (Söderberg et al., 2000). For the PP gene, only the exon containing most of the mature PP sequence, preceded by the signal peptide, could be identified. In chicken and mammals, the PYY and PP genes are located in tandem approximately 10 kb apart. The intergenic distance is of a similar magnitude or greater in the coelacanth (the genomic sequence has a gap in the assembly). All three peptide genes have a separate exon for 5′UTR in mammals and this exon has not been identified in any of the coelacanth genes.

## Discussion

**Figure 6:**
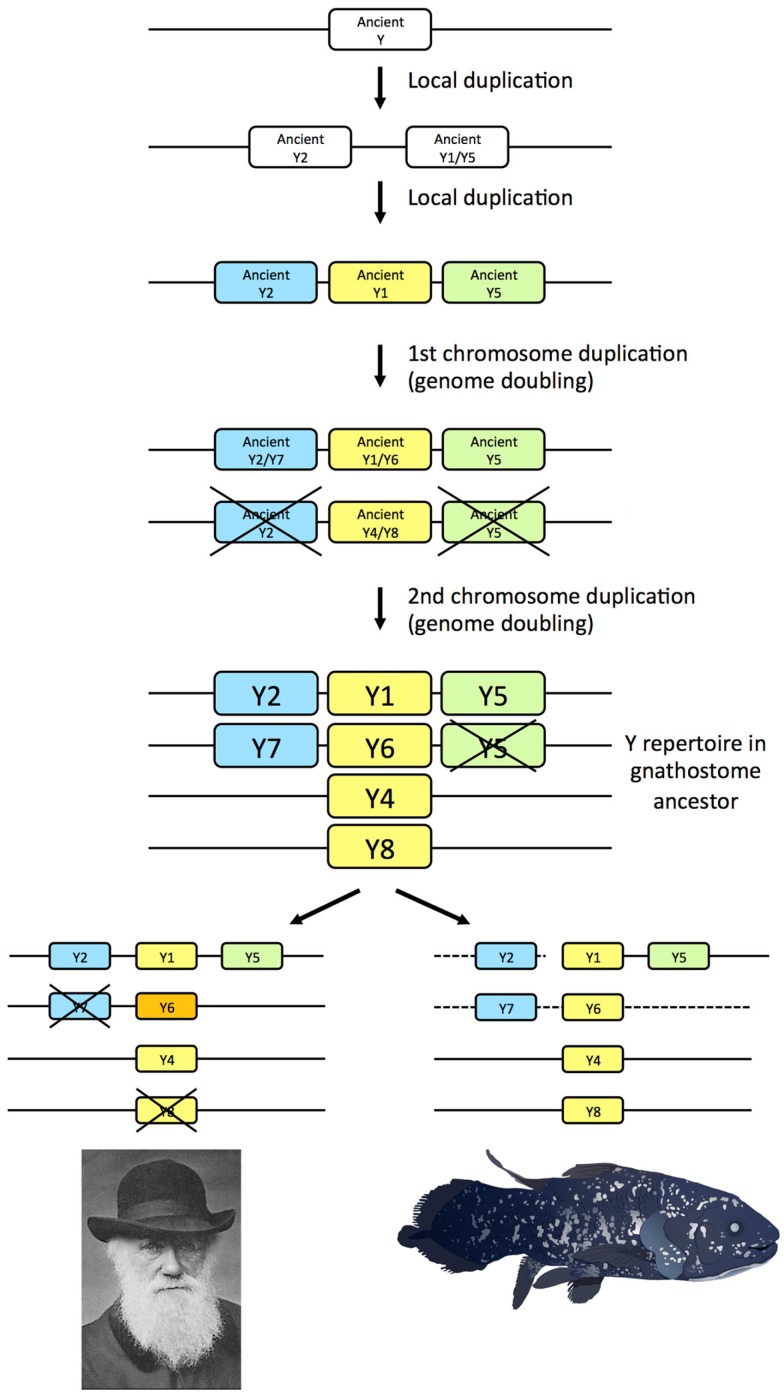
**Gene duplication events for the NPY receptor family in early vertebrate evolution including the two basal tetraploidizations**. Crosses mark gene losses. The human Y6 gene is a pseudogene, as indicated by orange color. The dashed lines for the coelacanth indicate that it is not known if the genes are syntenic.

The seven NPY-family receptor genes identified in the coelacanth genome database display orthology to the subtypes previously deduced to have arisen in the ancestor of the jawed vertebrates, namely subtypes Y1, Y2, Y4, Y5, Y6, Y7, and Y8 (Figure 3). Thus, the coelacanth has retained the complete ancestral repertoire like the elephant shark, but in contrast to mammals, chicken, and teleost fishes, all of which has lost one or more of the ancestral receptors. Our findings in the coelacanth corroborate our previous gene duplication scenario for the family of NPY receptors (Figure 6), with an ancestral local gene triplication, defining the three subfamilies of Y1, Y2, and Y5, followed by the chromosome quadruplication resulting from the two basal vertebrate tetraploidizations (Larhammar and Salaneck, 2004). Recent studies of other receptor families for neuroendocrine peptide-receptors have also revealed surprising ancestral vertebrate complexity, with six ancient somatostatin receptors (Ocampo Daza et al., 2012), six ancestral oxytocin-vasopressin receptors (Ocampo Daza et al., 2011; Yamaguchi et al., 2012; Ocampo Daza, 2013), and four ancestral GnRH (gonadotropin-releasing hormone) receptors (Kim et al., 2011), followed by losses of genes in mammals as well as other vertebrate lineages.

The clades in the NJ tree shown in Figure 2 corresponding to the seven receptor subtypes have high bootstrap support, except for the Y4–Y8 clade which often has been found to display poor resolution (Larsson et al., 2008, 2009) due to faster evolution of the Y4 sequences (Lundell et al., 2002) and the deviating amino acid composition of the zebrafish Y4 receptor, initially named Ya (Starbäck et al., 1999; see Salaneck et al., 2003). Analyses with the ML method give similar topology with this repertoire of species (not shown). Also when additional species are included for NJ and ML, these receptor subtypes fail to form completely separate clades with high bootstrap values, but the orthology relationships between tetrapods and teleosts have been confirmed by conserved synteny for Y4 and Y8 (Larsson et al., 2008). The seven coelacanth receptors can clearly be identified as orthologs of the previously identified seven ancestral vertebrate receptors, as shown by the tree in Figure 2.

Within each clade in Figure 2, some deviations from the established evolutionary relationships between species can be seen except Y7 which does indeed conform to the conventional species topology. As has been observed for several other gene or protein families, the zebrafish branch is more basal than the elephant shark, namely for Y1 and Y2. This is probably due to a higher evolutionary rate in the teleost fish lineage as compared to cartilaginous fishes and sarcopterygians, as has been observed for conserved non-coding elements (Lee et al., 2011). Also the mouse Y6 branching can probably be accounted for by its higher evolutionary rate. Analyses using the ML method displays similar minor deviations from the species relationships (not shown).

The coelacanth genome project has insufficient coverage for assembly into large scaffolds and therefore does not allow analyses of conserved synteny. Nevertheless, the Y1 and Y5 genes were found to be located close together in a head-to-head orientation like in mammals and chicken, with a similar distance as in human, supporting coregulation of the genes. Y1 and Y5 are known to be coexpressed in brain regions of rat (Parker and Herzog, 1998, 1999; Wolak et al., 2003) and mouse (Naveilhan et al., 1998; Oberto et al., 2007). In the paraventricular region of the hypothalamus, both Y1 and Y5 are known to contribute to the appetite-stimulating effect of NPY as reviewed in Mercer et al. (2011), probably in slightly different ways (Lecklin et al., 2002, 2003). However, the most important promoters and regulatory elements remain to be functionally identified. Indeed, comparison of the pig promoter regions (Wraith, 1999) with those in human (Herzog et al., 1997) suggested that multiple alternative start sites and/or 5′UTR exons exist that may differ even between these mammals, indicating complicated regulation of the two genes.

Based upon previous studies of the highly conserved peptides NPY and PYY, the identification of these in the coelacanth was expected and both of the mature peptides were found to be highly conserved. Indeed, the coelacanth PYY sequence appears to be identical to that of the deduced vertebrate ancestral PYY sequence (Larhammar, 1996), whereas mammalian PYY has undergone a few amino acid replacements. Possibly, the strong conservation of PYY in the coelacanth indicates a broader expression, like in teleost fishes and lamprey where PYY is expressed in both endocrine cells and neurons (Söderberg et al., 1994, 2000; Cerdá-Reverter et al., 2000; Kurokawa and Suzuki, 2002; Montpetit et al., 2005; Murashita et al., 2006, 2009; Wall and Volkoff, 2013), whereas mammalian PYY is almost exclusively expressed in endocrine cells. Broad distribution and/or usage has been found to correlate with higher conservation (Jordan et al., 2005; Khaitovich et al., 2005).

Our finding of PP in the coelacanth genome was unexpected because this peptide has previously only been identified in tetrapods (Larhammar, 1996; Cerdá-Reverter and Larhammar, 2000; Conlon, 2002; Sundstrom et al., 2008). The discovery of PP in the coelacanth could push back its origin by approximately 50 Myr, from a minimum of 340 Myr to a minimum of 390 Myr (Blair and Hedges, 2005). This may even raise the possibility that PP arose before the divergence of lobefinned fishes and rayfinned fishes, and that the PP gene has been lost in the rayfinned fish lineage. The NPY-family peptides so far described in teleosts have been assigned as NPYa, NPYb, PYYa, and PYYb (previously called PY), with the a and b duplicates having resulted from the teleost tetraploidization 3R (Sundstrom et al., 2008). PP in mammals binds to Y4 with higher affinity than either NPY or PYY, showing that partner preference has evolved between PP and Y4 in this lineage (Lundell et al., 1995, 1996; Berglund et al., 2001). In chicken, in contrast, all three peptides bind to Y4 with similar affinities (Lundell et al., 2002). Interestingly, Lch PP does not have the change from the ancestral residue Gln-34 to Pro-34 that is found in all PP sequences, except a few bird sequences that have a histidine at this position and the caecilian *Typhlonectes natans* (an amphibian) which has a serine (Conlon et al., 1998; Conlon, 2002). The coelacanth has retained the ancestral Gln-34 found also in all NPY and PYY sequences. This may indicate that Lch PP has a binding profile that differs from PP in tetrapods with regard to receptor subtype preferences.

Detailed studies of peptide-receptor interactions will require cloning and functional expression of the coelacanth receptors and synthesis of the peptides, especially Lch PP. Hopefully future crystallization of NPY-family receptors will provide good templates for structural modeling of the coelacanth receptors and thereby help explain how this peptide-receptor system has evolved in the vertebrates. Unfortunately, studies of the tissue distribution of the mRNA for the NPY-family peptides and receptors cannot be easily performed with this endangered species.

The large repertoire of NPY-family receptors in the coelacanth may either mean that it has retained the ancestral functions for the seven vertebrate receptors or evolved novel functions for some of them. If ancestral functions are maintained, this might give clues to what functions may have disappeared in mammals with the loss of receptors Y7 and Y8, as well as Y6 in several mammalian lineages. Another possibility is that any such functions are partially or completely mediated by the four to five receptors that still exist in mammals. Information on such scenarios may be obtained from studies of species that represent additional basally diverging lineages, such as lungfishes (which unfortunately have very large genomes, thereby complicating bioinformatic analyses) and basal actinopterygians like the spotted gar whose genome was recently assembled (Amores et al., 2011). This species is especially interesting as it diverged before the third tetraploidization took place in the teleost lineage. Also basal lineages among the teleosts have recently added valuable information on gene family evolution, especially the recently assembled genome for the European eel (Henkel et al., 2012). Clearly considerable genetic complexity arose at an early stage in vertebrate evolution that can be deduced by comparing distantly related vertebrate lineages. An inevitable conclusion is that for several gene families, mammals must be considered to have degenerated by gene loss.

## Conflict of Interest Statement

The authors declare that the research was conducted in the absence of any commercial or financial relationships that could be construed as a potential conflict of interest.
